# Characteristics of aggressive incidents in emergency primary health care described by the Staff Observation Aggression Scale – Revised Emergency (SOAS-RE)

**DOI:** 10.1186/s12913-019-4856-9

**Published:** 2020-01-13

**Authors:** Grethe E. Johnsen, Tone Morken, Valborg Baste, Knut Rypdal, Tom Palmstierna, Ingrid Hjulstad Johansen

**Affiliations:** 1 National Centre for Emergency Primary Health Care, NORCE Norwegian Research Centre, Kalfarveien 31, 5018 Bergen, Norway; 2 Centre for Research and Education in Forensic Psychiatry, Haukeland University Hospital, Bergen, Norway; 3 Centre for Psychiatry Research, Department of Clinical Neuroscience, Karolinska Institutet, Stockholm, Sweden

**Keywords:** Aggression, Workplace violence, Emergency primary health care

## Abstract

**Background:**

Workplace violence in emergency primary health care is prevalent, but longitudinal studies using validated assessment scales to describe the characteristics of workplace violence in these settings are lacking. The aim of the present study was to determine the characteristics of aggressive incidents in emergency primary health care clinics in Norway.

**Methods:**

Incidents of workplace violence were reported with the Staff Observation Aggression Scale – Revised Emergency (SOAS-RE). The study was conducted in ten emergency primary health care clinics over a period of one year.

**Results:**

A total of 320 aggressive incidents were registered. The mean overall SOAS-RE score for reported aggressive incidents was 9.7 on a scale from 0 to 22, and 60% of the incidents were considered severe. Incidents of verbal aggression accounted for 31.6% of all reported incidents, threats accounted for 24.7%, and physical aggression accounted for 43.7%. Verbal aggression was most often provoked by long waiting time. Physical aggression was most often provoked when the patient had to go through an involuntary assessment of health condition. Almost one third of the aggressors were females, and nurses were the most frequent targets of all aggression types. No differences in psychological stress were found between types of aggression.

**Conclusions:**

This study shows that workplace violence in emergency primary health care clinics is a severe problem. Patterns in provocation and consequences of aggressive incidents can be used to improve our understanding of and prevention and follow-up procedures of such incidents.

## Background

Workplace violence spans a spectrum from verbal aggression to physically violent acts and is a threat to the safety and wellbeing of health care workers and patients. It also negatively impacts on the ability to perform professional medical work. Research has shown that health care workers in emergency primary health care services are exposed to considerable verbal aggression, threats, and violence from patients and/or visitors [1].

The characteristics of aggressive incidents in psychiatric wards have been thoroughly documented [2–4]. However, studies of the characteristics of aggressive incidents in emergency primary health care are sparse. In order to expand our understanding of the aggressive incidents within primary health care, there is a need for structured measurement where different aspects of the aggressive behaviour are examined.

Evidence-based assessment procedures have been developed for measuring aggressive incidents in health care settings [5–7], and the Staff Observation Aggression Scale – Revised (SOAS-R) is a widely used and valid instrument developed for monitoring aggressive incidents in psychiatric wards [8]. Due to differences between emergency primary health care and psychiatric hospital settings, the SOAS-R needed adjustments and validation in order to be applied in emergency primary health care [9]. The new and validated instrument was called the Staff Observation Aggression Scale – Revised Emergency (SOAS-RE).

Emergency primary health care in Norway provides easily accessible care for an unselected patient population. The services are organised in emergency primary health clinics or as part of a general practitioner’s surgery. The clinics are staffed mainly by general practitioners and nurses, and the number of staff depends on the location and size of the population being served. The services provide care for a wide range of clinical conditions, and they forward the patients to inpatient care at somatic and psychiatric hospitals (secondary care) when needed. However, most of the patients receive all of their necessary treatments at the clinics.

To our knowledge, no previous studies have used forms developed for emergency primary health care clinics when measuring workplace violence. The forms used in impatient settings have limited relevance in outpatient settings, due to differences in clinical scenarios and service organizations. When examining workplace violence in outpatient clinics, it is necessary to take into account that these services provide easily accessible and unscheduled care for an unselected and largely unfamiliar patient population. With using a scale developed for emergency primary health care we expect to find characteristics of aggressive incidents in these settings. These findings can be used in the work of improving service provision in emergency primary health care.

The aim of this study was to describe the characteristics of aggressive incidents in emergency primary health care clinics in Norway and to estimate the severity of such incidents. Further aims were to describe differences in incidents based on type of aggression (verbal aggression and threats versus physical aggression) and to determine if the severity of the incident was related to what time of day the incident occurred.

## Methods

### Instrument

The emergency primary health care version of the Staff Observation Scale was developed for this study by adapting the Staff Observation Scale - Revised [10] into the Staff Observation Aggression Scale – Revised Emergency (SOAS-RE). The adjustment and validation of the scale have been thoroughly reported elsewhere [9].

The SOAS-RE report form consists of six columns (categories) to be completed each time a staff member is involved in an aggressive incident. Each of the columns in the report form represents a time-wise aspect of the aggressive incidents. Each column includes predefined alternatives to be ticked off when describing the actual incident. The six columns are recorded as follows: 1) The provocation that lead to the aggressive incident, e.g. “the patient had to wait”; 2) The means used by the patient during the incident, e.g. “verbal aggression” or “hand”; 3) The target of the aggression, e.g. “objects” or “patients”; 4) The consequences for victims, e.g. “pain” or “needed treatment by a physician”; 5) The measures taken to stop or control the aggressive behaviour, e.g. “talking to the patient” or “restraining the patient by force”; and 6) Persons involved in stopping the aggression, e.g. “physician”, “nurse”, or “police”. Each factor has a score, and the highest score from each column is added to give a total score. Just like the original SOAS-R, the total SOAS-RE severity score ranges from 0 to 22 points. An SOAS-RE severity score of 9 or more is judged to be a severe incident, which is the score used to identify severe incidents when using the SOAS-R. Such incidents include all physical attacks causing fear or harm to the victims, as well as attacks against persons using dangerous objects [9]. In addition to marking the relevant factors in the six columns, the worker marks the severity of the aggression on a 100 mm Visual Analogue Scale (VAS) ranging from “not severe at all” (at the 0 end of the VAS) to “extremely severe” (at the 100 end of the VAS). The form also records background information about where the incident took place (clinic, phone, home visit), the worker’s age, occupation, and gender, and information about the aggressor’s gender.

A total of 350 forms were collected during the study period. In 24 incidents more than one health care worker was involved, and in 21 incidents two individuals filled in separate forms and in 3 incidents three, four, and five individuals, respectively, filled in separate forms. For each incident with several forms, one form was chosen using a random number generator (https://www.random.org/) in order to ensure that the remaining incidents were independent.

### Sample description

This study was conducted in 11 emergency primary health care clinics from nine different counties in Norway. The clinics self-recruited for the study. One clinic did not forward any forms during the study period, and thus the included incidents were reported from 10 clinics covering a population of 1.3 million inhabitants. The observation period was January to December of 2016.

### Procedure

Before the initiation of the study, all of the included emergency primary health care clinics were visited by one of the researchers. This researcher gave verbal and written instructions to the contact person in how to take part in the study. They were informed that after an aggressive incident, the emergency primary care worker involved in the aggressive situation was supposed to complete the SOAS-RE, including the VAS. The anonymously completed forms were received by the contact person at the clinic and forwarded to the National Centre for Emergency Primary Health Care.

### Statistical analyses

The analyses were performed using SPSS Statistics V.25. Descriptive statistics, including mean and standard deviation (SD), were calculated for the general overview of the characteristics of the aggressive patients and visitors and the reporting nurses and physicians. In order to examine differences based on type of aggression, the answers in the column ‘means used by aggressor’ were re-coded into three categories dependent on the most severe aggression reported; (1) verbal aggression exclusively, (2) threats, and (3) physical aggression (where one or more of the factors regarding “ordinary objects”, “parts of the body”, or “dangerous objects or methods” were marked to describe the means used by the aggressor in the actual incident). Four incidents could not be defined and were set to missing in the analysis of type of aggression. Differences in event characteristics were examined by aggression type (verbal only, threats, and physical aggression), and possible differences between types of aggression were analysed with chi-square tests or ANOVA. Statistical significance was set to *p* < 0.05.

## Results

During the study period, 320 individual incidents of aggression were reported from the participating emergency primary health clinics. As shown in Table 1, aggressive incidents were most frequently reported during evening and night shifts. Younger health care workers reported 44.4% of the aggressive incidents (mean age 34.1 years, SD = 9.5), and 71.5% of the incidents were reported by females. About a third (28.2%) of the aggressors were female.

**Table 1:** Site, time and characteristics of the persons involved in the aggressive incidents (*n* = 320)

	n^a^	%
Time of incident		
Day of week		
Monday - Friday	201	62.8
Saturday - Sunday/public holidays	119	37.2
Shift		
Day	75	25.2
Evening	113	37.9
Night	110	36.9
Season		
Spring	92	28.7
Summer	62	19.4
Fall	51	15.9
Winter	115	35.9
Characteristics of health worker involved		
Age		
21–30 years	131	44.4
31–40 years	98	33.2
41–50 years	40	13.6
> 51	26	8.8
Gender		
Female	218	71.5
Male	87	28.5
Occupation		
Nurse	254	82.2
Physician	41	13.3
Other	14	4.5
Characteristics of aggressor		
Gender		
Female	73	28.2
Male	181	69.9
Several aggressors	5	1.9
Relation to health service		
Patient	246	83.7
Next of kin	44	15.0
Both patient and next of kin	3	1.0

^a^Due to missing data on some items, it does not add up to the total of 320

Verbal aggression was reported in 250 of the 316 incidents (79.1%). Exclusively verbal aggression was reported in 31.6% (*n* = 100) of the events, threats were reported in 24.7% (*n* = 78) of the events, and physical aggression was reported in 43.7% (*n* = 138) of the events.

Among the physical aggressive incidents, 66.7% also involved verbal aggression and 32.6% also involved threats, which means that only one incident involved only physical aggression. In performing physical aggression, the aggressors mostly used body parts (81.2%). Dangerous objects and methods (e.g., weapon, knife, or syringe) were used in 11.6% of the physically aggressive incidents (*n* = 16), and ordinary objects were used in 28.3% of the incidents. Physicians reported the most threats (33.3%) and physical aggression (53.8%), while nurses reported fewer threats (23.4%) and less physical aggression (41.7%) (*p* = 0.022).

There was no obvious provocation in 27.5% (*n* = 87) of the incidents (Table 2). Waiting time was a more frequent provocation among the exclusively verbally aggressive incidents (39.0%) compared to threats (26.9%) and physically aggressive incidents (9.4%) (*p* < 0.001). To be denied something was a more frequent provocation among the incidents of threats (30.8%) than among verbally or physically aggressive incidents, and there was a higher proportion of involuntary assessments of health condition (12.3%) and other provocations (29.7%) among physically aggressive incidents compared to verbal aggression and threats.

**Table 2:** SOAS-RE aggressive incidents by type of aggression (verbal, threats, physical) and total

	Type of aggression								
	Verbal aggression		Threats		Physical aggression			Total	
	*(n* = 100)		(*n* = 78)		(*n* = 138)			(*n* = 316)	
SOAS-RE columns^1^	n	(%)	n	(%)	n	(%)	*p*-value^2^	n	(%)
Provocation of aggressive behavior									
No understandable provocation	24	(24.0)	22	(28.2)	41	(29.7)	0.615	87	(27.5)
Person had to wait	39	(39.0)	21	(26.9)	13	(9.4)	< 0.001	73	(23.1)
The person was denied something	25	(25.0)	24	(30.8)	21	(15.2)	0.022	70	(22.2)
The person disagreed about assessment/advice	31	(31.0)	25	(32.1)	27	(19.7)	0.063	83	(26.3)
Involuntary assessment of health condition		(3.0)	3	(3.8)	17	(12.3)	0.010	23	(7.3)
Other	10	(10.0)	9	(11.5)	41	(29.7)	< 0.001	60	(19.0)
Target of aggression									
None	2	(2.0)	0		2	(1.4)		4	(1.3)
Furniture/object	1	(1.0)	4	(5.1)	26	(18.8)	< 0.001	31	(9.8)
Physician	17	(17.0)	23	(29.5)	33	(23.9)	0.140	73	(23.1)
Nurse	84	(84.0)	62	(79.5)	104	(75.4)	0.269	250	(79.1)
Ambulance personnel	3	(3.0)	4	(5.1)	9	(6.5)	0.473	16	(5.1)
Security guard/police	5	(5.0)	4	(5.2)	25	(18.1)	0.001	34	(10.8)
Other patients/persons	12	(12.0)	11	(14.1)	13	(9.4)	0.567	36	(11.4)
Consequence(s) for victim(s)									
None	36	(36.0)	29	(37.2)	42	(30.4)	0.519	107	(33.9)
Object(s) damaged	0		1	(1.3)	13	(9.4)		14	(4.4)
Psychological stress	43	(43.0)	34	(43.6)	67	(48.6)	0.643	144	(45.6)
Felt threatened	28	(28.0)	30	(38.5)	72	(52.2)	0.001	130	(41.1)
Pain < 10 min	0		1	(1.3)	10	(7.2)		11	(3.5)
Pain > 10 min	0		0		4	(2.9)		4	(1.3)
Visible injury	0		0		4	(2.9)		4	(1.3)
Need for treatment by a physician	0		1	(1.3)	2	(1.4)		3	(0.9)
Need to be taken off duty	3	(3.0)	3	(3.8)	9	(6.5)		15	(4.7)
Other	3	(3.0)	0		8	(5.8)		11	(3.5)
Measure(s) to stop aggression									
None	5	(5.0)	1	(1.3)	4	(2.9)	0.383^3^	10	(3.2)
Talked to the person	67	(67.0)	49	(62.8)	63	(45.7)	0.002	179	(56.6)
Took the person aside	5	(5.0)	5	(6.4)	16	(11.6)	0.150	26	(8.2)
Withdrew for situation/ended call	30	(30.0)	25	(32.1)	48	(34.8)	0.734	103	(32.6)
Complied with persons wish	1	(1.0)	5	(6.4)	1	(0.7)	0.019^3^	7	(2.2)
Asked the person to leave the site	6	(6.0)	14	(17.9)	22	(15.9)	0.031	42	(13.3)
Forced the person to leave/held by force	8	(8.0)	6	(7.7)	45	(32.6)	< 0.001	59	(18.7)
Other	17	(17.0)	14	(17.9)	41	(29.7)	0.035	72	(22.8)
Persons involved in measure(s) to stop aggression									
Physician	13	(13.0)	26	(33.3)	33	(23.9)	0.005	72	(22.8)
Nurse	78	(78.0)	50	(64.1)	91	(65.9)	0.071	219	(69.3)
Ambulance personnel	7	(7.0)	8	(10.3)	12	(8.7)	0.740	27	(8.5)
Security guard/police	25	(25.0)	28	(35.9)	93	(67.4)	< 0.001	146	(46.2)
Other patients	0		0		1	(0.7)		1	(0.3)
Next of kin	0		4	(5.1)	10	(7.2)		14	(4.4)
Other	6	(6.0)	8	(10.3)	7	(5.1)	0.324	21	(6.6)

^1^ Multiple responses were possible in each SOAS-RE column

^2^ *p*-value from a Chi-square test for differences between type of aggression, omitted if too few cases

^3^ Exact test

Nurses were the most frequent targets of all aggression types (79.1%), and physicians were the second most frequent (23.1%) (Table 2). In 11.4% (*n* = 36) of all incidents, patients or other visitors were the targets. Security guards and police were the targets in 18.1% (*n* = 25) of the incidents of physical aggression.

Victims described psychological stress in 45.6% of the incidents, and there were no significant differences between the aggression types (Table 2). The victims felt more threatened in incidents involving physical aggression (*p* = 0.001). In 4.8% (*n* = 15) of the incidents the victims experienced pain, and in 4.7% of the incidents the victims had to be taken off duty.

Talking to the patient was the most frequently used measure to resolve the situation (Table 2). Restrictive measures such as to forcing the person to leave or restraining the person by force were used in 32.6% (*n* = 45) of the physically aggressive incidents. In only 2.2% (*n* = 7) of the incidents did health care personnel comply with the patients’ wishes in order to stop the violence.

There was a higher frequency of physicians (33.3%) involved in stopping incidents of threats compared to physicians being involved in stopping physically or verbally aggressive incidents (23.9 and 13.0%, respectively) (Table 2). Security guards or police were most frequently involved in stopping physical aggression, and this was different from incidents of verbal aggression and threats (*p* < 0.001). Generally, incidents of physical aggression involved more people than incidents of verbal aggression or threats.

The severity score of the reported events on the SOAS-RE ranged from 0 to 21, with a mean score of 9.7 (SD = 4.2). With a cut-off score of ≥9, 60% (*n* = 189) of the incidents were considered severe. The severity of the SOAS-RE mean score for exclusively verbal incidents was 7.1 (SD = 3.4), for threats it was 9.2 (SD = 3.4), and for physical incidents it was 12.0 (SD = 4.0) (*p* < 0.001). The mean VAS score was 44.6 (SD = 26.0). The mean VAS score for exclusively verbal incidents was 31.0 (SD = 20.5), for threats it was 47.1 (SD = 25.9), and for physical aggression it was 54.0 (SD = 25.5) (*p* < 0.001).

Table 3 shows the severity in SOAS-RE and VAS scores by day of week, shift, and season. Comparison of the day of the week or shifts revealed no significant differences in severity. When it comes to seasons however, we fount higher SOAS-RE severity scores in the summer season (*p* = 0.033).

**Table 3:** Severity of SOAS-RE and VAS score by time of incident

	SOAS-RE			VAS		
	Mean	SD	*p*-value	Mean	SD	*p*-value
Day of week						
Monday - Friday	9.6	4.1		43.1	25.6	
Saturday - Sunday/public holidays	9.9	4.5	0.469	47.3	26.6	0.196
Shift						
Day	8.9	4.5		44.4	28.5	
Evening	9.5	4.1		44.6	24.5	
Night	10.3	4.1	0.058	44.7	24.7	0.997
Season						
Spring	10.1	4.5		47.3	26.7	
Summer	10.6	4.2		42.8	23.2	
Fall	9.7	4.0		47.1	27.2	
Winter	8.8	4.0	0.033	42.1	26.0	0.448

## Discussion

This study shows that in many cases the reported workplace violence in Norwegian emergency primary health care is severe. The most frequently reported aggressive behaviour was verbal aggression (79%), and this was most frequently provoked by waiting time. However, verbal aggression alone was reported in only one third of the incidents. Nearly all reported aggressive incidents provoked by involuntary assessment of the patients’ health condition included physical aggression. Most aggressors were males, but almost one third were females. Independent of type of aggression, talking to the aggressor was the most common measure to stop the aggression. Psychological stress, as a consequence for the victims, was reported in all types of aggressive incidents.

### Comparisons with other studies, possible explanations, and implications

The overall mean severity score of the SOAS-RE (9.7) in the present study is in line with previous studies with SOAS-R conducted on inpatients in psychiatric hospitals where the severity scores ranged from 9.2 to 11.0 [8]. Aggressive incidents in emergency primary care are thus as severe as those reported from psychiatric and psychogeriatric wards. This finding is not surprising given the diversity of patients attending Norwegian emergency primary health care clinics, the clinics’ obligation to assess all attending patients, and the strict two-tiered health care system in which these clinics have a gatekeeper function for both somatic and psychiatric conditions.

Several studies have found that verbal aggression is the most common type of workplace violence among health care personnel [11, 12], and our results confirm this. Also, our study shows that physical aggression and threats frequently coexisted with verbal aggression. The high proportion of physical aggression (43%) might reflect that such incidents are more likely to be reported. Underreporting of verbal aggression has been documented in previous studies, and this might have influenced our results [13, 14]. Comparing results from studies on workplace violence is also difficult because the definition of violence and the categories of workplace violence vary between studies. Further studies using evidence-based clinical scales such as the SOAS-RE would improve our ability to compare results between studies.

Our study indicates that waiting time is a central provocation for verbal aggression, and other studies in emergency departments in hospitals have also found waiting times to be a precipitating factor for violence [12, 15]. In Norway, emergency primary health care services are characterised by high contact rates by telephone, and in most districts the only way to contact health care services is by calling first. When calling, nurses triage the patients’ needs and schedule appointments at the clinic. However, many large city clinics have not yet implemented a telephone triage system. Our results suggest that reducing the time patients spend waiting in the clinic might be one way to reduce verbal aggression. Nearly all reported aggressive incidents with involuntary assessment as the provoking factor included physical aggression. This confirms previous qualitative findings that have identified involuntary assessment of the patients as a high-risk situation for workplace violence [16]. Involuntary assessment must therefore be recognised as a risk situation were the likelihood for physical aggression is high, and awareness of this risk situation should be in focus as part of the safety training of health care personnel.

Nurses were the most frequent targets for all types of aggression. Several studies have found nurses to be at greatest risk of workplace violence [17, 18], and these findings have been explained by the nurses’ frontline position in which they operate the phone and reception desk and interact with patients for longer periods of time. This finding might also be influenced by the ratio between nurses and physicians. Few studies have, however, compared nurses and physicians’ different exposures to different types of aggression. In our study, nurses reported more verbal aggression incidents than physicians, and physicians reported more threats and physical aggression incidents than nurses. These findings indicate that the physicians’ professional role in making decisions regarding treatment might induce conflicts that elicit threats and/or physical violence. The propensity to report verbal aggression might also differ between the professions.

In accordance with previous research conducted in emergency departments, our study shows that the patients are the main aggressors [19]. Next of kin, however, might also be aggressors. Qualitative research has shown that unmet needs might lead to frustrations, fear, and aggression in both patients and relatives [16]. Expectations and unmet needs might be related to a mismatch between the patient’s expectations and the services offered at emergency primary health care. One way to resolve this is by consistent communication to the public regarding proper use of the health care service and thereby clarifying the role of the emergency primary health clinic.

Similar to emergency department studies, most aggressors were males [20]. However, in our study the proportion of female aggressors was higher than reported in previous emergency department studies. Discrepancies between cultures, countries, and health sectors might explain these differences.

VAS severity scores showed significant differences between aggression types. However, our study found no significant differences between type of aggression and reporting of psychological stress, indicating that all types of aggression might be stressful for the health care personnel involved. Further, there were no significant differences in the need to be taken off duty between threats and physical aggression, confirming the seriousness of such threats. This implies that all types of aggression need to be taken seriously and that every incident should be managed and followed up independently of the type of aggression reported.

Situations where no obvious provocations were mentioned were distributed equally between verbal aggression, threats, and physical aggression. The use of alarm systems is essential for the management of physical aggression that occurs unexpectedly. However, the existence of alarms is no guarantee for safety, and our results emphasise the importance of short response times to alarms. Previous research has found increasing preparedness through training and management of violence [21–23], and the unexpectedness of the aggression found in this study further underlines this. Training might be one strategy to increase health care personnel’s awareness of such risk situations.

More incidents occurred on evening and night shifts, but there were no differences in severity depending on the time of day of the incident. When considering that night shifts in these services generally have few contacts (about 12.2% of all contacts occur during night shifts) [24], the number of aggressive incidents on night shifts is high. However, other studies in emergency departments have shown contrasting results. Crilly et al. [12] found that most verbal and physical incidents in emergency departments occurred on evening shifts. Kowalenko et al. [25] on the other hand found that time of day had no influence on incidence of violence. To our knowledge, studies conducted in emergency departments have not incorporated severity measures that take into consideration the time of day. More incidents occurred during the spring and the winter seasons. The summer season had a more serious SOAS-RE severity scores. It could be related to that the summer season is a holiday period, and that the clinics during summer is staffed by more temporary and unexperienced health care personnel on duty.

### Strengths and limitations of the study

The strength of this study is that we have examined workplace violence using a validated instrument that measures different aspects of violence (provocation, means used, target, consequences, etc.). The scale is easy to complete and can be used as an instrument for incident reporting and statistics in emergency primary health care clinics and as a supplement to official injury reports. The SOAS-RE offers standardised classifications of aggressive events and has a scale-based scoring system to identify severity, and this eliminates some of the subjectivity in the reporting. Participating emergency primary health care clinics were recruited among a broad geographical distribution and of different sizes and with different organisational assets. The results are thus likely to be representative of Norwegian emergency primary health care. Underreporting of verbal aggression or low-level violence has been reported in other studies using the SOAS-R, and it is possible that these incidents are not fully reported in our study [8]. Further studies are needed to clarify whether this mainly reflects the threshold of reporting violence or whether it is a real difference in the incidents that occur. The total number of incidents in our study was relatively small, and future larger studies are needed to replicate the current findings. If verbal aggression is indeed overlooked or ignored and not reported, studies using qualitative designs might be one way to further explore this.

## Conclusions

The present study is an important step to gaining an in-depth understanding of the everyday violence that health care personnel in emergency primary health care clinics are exposed to. Our findings reveal that the aggression that health care personnel experience is often severe, that verbal and physical aggression are provoked by different factors, and that reports of psychological stress do not significantly differ between types of aggression. The SOAS-RE advantage is that it can be used to register incidents, identify challenges, give indicators on how to prevent and manage violent episodes. Our results indicate that one has to consider human relations, sufficient staffing and physical structures to ensure safe working conditions in emergency primary health care. Future studies should measure incident rates of aggressive events in emergency primary health care clinics and should explore the psychological stress that nurses and physicians experience from such events.

## Data Availability

The dataset analysed in the present study is available from the corresponding author on reasonable request.

## Abbreviations

SD: Standard deviation
SOAS-R: The Staff Observation Aggression Scale – Revised
SOAS-RE: The Staff Observation Aggression Scale – Revised Emergency
VAS: Visual Analogue Scale
